# Age-related alterations in the cardiovascular responses to acute exercise in males and females: role of the exercise pressor reflex

**DOI:** 10.3389/fphys.2023.1287392

**Published:** 2023-11-02

**Authors:** A. K. Grotle, J. V. Langlo, E. Holsbrekken, A. J. Stone, H. Tanaka, P. J. Fadel

**Affiliations:** ^1^ Department of Sports, Food and Natural Science, Western Norway University of Applied Sciences, Bergen, Norway; ^2^ Department of Kinesiology and Health Education, The University of Texas at Austin, Austin, TX, United States; ^3^ Department of Kinesiology, The University of Texas at Arlington, Arlington, TX, United States

**Keywords:** blood pressure, sympathetic nerve activity, metaboreflex, mechanoreflex, group III and IV muscle afferents, autonomic control

## Abstract

Autonomic adjustments of the cardiovascular system are critical for initiating and sustaining exercise by facilitating the redistribution of blood flow and oxygen delivery to meet the metabolic demands of the active skeletal muscle. Afferent feedback from active skeletal muscles evokes reflex increases in sympathetic nerve activity and blood pressure (BP) (i.e., exercise pressor reflex) and contributes importantly to these primary neurovascular adjustments to exercise. When altered, this reflex contributes significantly to the exaggerated sympathetic and BP response to exercise observed in many cardiovascular-related diseases, highlighting the importance of examining the reflex and its underlying mechanism(s). A leading risk factor for the pathogenesis of cardiovascular disease in both males and females is aging. Although regular exercise is an effective strategy for mitigating the health burden of aging, older adults face a greater risk of experiencing an exaggerated cardiovascular response to exercise. However, the role of aging in mediating the exercise pressor reflex remains highly controversial, as conflicting findings have been reported. This review aims to provide a brief overview of the current understanding of the influence of aging on cardiovascular responses to exercise, focusing on the role of the exercise pressor reflex and proposing future directions for research. We reason that this review will serve as a resource for health professionals and researchers to stimulate a renewed interest in this critical area.

## Introduction

Aging adversely affects cardiovascular health, contributing to an increased risk of developing and dying from cardiovascular disease (CVD) with each decade of life in both males and females (Lindstrom et al., 2022). Indeed, a recent report from the American Heart Association suggests that the prevalence of CVD progressively rises across the lifespan, from −40% among adults aged 40–59 years to around 75% in those aged 60–79 years, and further surging to 86% in those 80 years and above (Tsao et al., 2022). Impaired blood pressure (BP) regulation, as evidenced by high BP, is a leading contributor to enhanced CVD burden and premature death with aging. Notably, the prevalence of elevated BP in adults (30–79 years) has nearly doubled in the last 2 decades and affects about 60% of people aged 60 years and above (Williams et al., 2018). Importantly, with the dramatic and continued growth of the proportion of people 65 and older, the health and financial burden of age-related cardiovascular dysfunction will undoubtedly magnify in the future (Roth et al., 2015; Roth et al., 2020).

Epidemiological evidence suggests that healthy older adults are more likely to experience an exaggerated BP response (e.g., peak change in systolic, diastolic, or mean BP) to sub-maximal and maximal large-muscle mass dynamic exercise when compared to healthy young adults (Stratton et al., 1994; Daida et al., 1996; Sabbahi et al., 2018). Furthermore, this response is also observed in smaller cohort studies comparing normotensive and exercise-trained healthy young and older individuals (Ogawa et al., 1992). It is well-accepted that an exaggerated BP response to exercise has a prognostic value, indicating a higher risk of sudden cardiac death, myocardial infarction, future hypertension, CVD, and left ventricular hypertrophy (Wilson and Meyer, 1981; Alderman et al., 1990; Mundal et al., 1996; Barlow et al., 2014). Notably, an exaggerated exercise BP also has prognostic value in normotensive and highly fit individuals (Caselli et al., 2019). Most importantly, anti-hypertensive medications appear ineffective in reducing an exaggerated BP response to exercise (Chant et al., 2018). Therefore, understanding the underlying mechanisms responsible for this response is imperative.

Although multifactorial, alterations in autonomic nervous system regulation likely play a role. Indeed, appropriate autonomic adjustments of the circulation mediated by feedforward signals from higher brain centers (i.e., central command) and feedback signals from the contracting skeletal muscle (i.e., exercise pressor reflex) are vital to increasing BP and matching metabolic demand and supply in the contracting skeletal muscles (Alam and Smirk, 1937; Goodwin et al., 1972; Mark et al., 1985). In recent years, considerable attention has been given to the exercise pressor reflex, as alterations in this reflex play a major role in mediating an exaggerated BP response to exercise observed in many age-related diseases (Piepoli et al., 1996; Baccelli et al., 1999; Leal et al., 2008; Park et al., 2008; Tsuchimochi et al., 2010; Holwerda et al., 2016; Grotle et al., 2019).

Due to conflicting findings, the effects of aging, independent of pathology and physical activity levels, on the exercise pressor reflex are unclear and controversial. Indeed, reports have suggested that the BP response to reflex activation is blunted (Markel et al., 2003), preserved (Ng et al., 1994; Momen et al., 2004; Houssiere et al., 2006; Roseguini et al., 2007; Greaney et al., 2013; Sidhu et al., 2015), or exaggerated (Choi et al., 2012; Schneider et al., 2018; Hasegawa et al., 2021; Wenner et al., 2022). Thus, this review aims to address these discrepant findings and provide a brief overview of the current understanding of the influence of aging on the cardiovascular responses to exercise, focusing on the role of the exercise pressor reflex and proposing future directions for this critical area of research.

## Cardiovascular adjustments to exercise: role of the exercise pressor reflex

Transitioning from rest to exercise dramatically increases skeletal muscle metabolic activity, causing an immense integrative challenge. The body meets this challenge primarily through cardiovascular adjustments mediated by neural control mechanisms. These mechanisms mainly include feedforward signals evoked by central command and feedback signals evoked by the exercise pressor reflex. Moreover, resetting the arterial baroreflex allows continued regulation of BP during exercise (Melcher and Donald, 1981; Fadel and Raven, 2012). Acknowledging that central command and arterial baroreflexes also play an important role during exercise is essential. However, we focus on the exercise pressor reflex due to the limited space and evidence on the age effect and interaction with the exercise pressor reflex.

The exercise pressor reflex is defined as “all the cardiovascular changes reflexively induced from contracting skeletal muscle that is responsible for the increase in arterial BP” (Mitchell et al., 1983). In brief, mechanical deformation and metabolic products from muscle contraction activate channels and receptors on the peripheral nerve endings, increasing the discharge frequency of group III and IV muscle afferents (Coote et al., 1971; Kaufman et al., 1983; Rotto and Kaufman, 1988; Amann et al., 2010). These afferents project centrally via the dorsal horn of the spinal cord to the brainstem, where complex interaction with other inputs (e.g., arterial baroreflex and central command) occur in neuroanatomical pathways involving the nucleus tractus solitarii and lateral reticular nucleus located in the medulla oblongata (Iwamoto et al., 1982; Potts, 2001). The resultant efferent reflex action is decreased parasympathetic nerve activity to the heart and increased sympathetic nerve activity to the heart and peripheral blood vessels, facilitating an intensity-dependent increase in cardiac output and BP (Mark et al., 1985; Cui et al., 2006; Amann et al., 2010). Both mechanical and metabolic stimuli, alone or in combination, are effective in evoking reflexive increases in BP, with these two components commonly referred to as the mechanoreflex and metaboreflex, respectively. (Kaufman et al., 1983; Kaufman and Hayes, 2002). However, it is critical to note that group III and IV afferents exhibit polymodal activity (Rotto and Kaufman, 1988; Rotto et al., 1990). For further detail on this reflex mechanism in health and disease, as well as the specific stimuli and their corresponding receptor and channels resulting in activation of the mechanoreflex and metaboreflex, we refer readers to recent detailed reviews (Grotle et al., 2020; Teixeira and Vianna, 2022).

Various protocols are utilized to study the exercise pressor reflex and its two components in humans and animals. Blood flow occlusion to enhance or trap the metabolic stimuli during or after exercise (post-exercise ischemia; PEI), is a standard procedure to isolate the metaboreflex component. Passive movement or stretching of the skeletal muscles or rhythmic contractions provides a robust mechanical stimulus without substantial production of metabolites. It is, therefore, commonly used to assess the mechanoreflex component. It is worth noting that studying the complete exercise pressor reflex (mechanoreflex + metaboreflex), independent of central command, is difficult in humans and requires sophisticated invasive techniques. In this regard, the current approach is to compare BP responses before and after the injection of lumbar intrathecal fentanyl, which temporarily attenuates muscle afferent feedback from the contracting muscle while preserving force-generating capacity.

## Effects of aging on the blood pressure response to exercise

It is fairly well-described in the literature that older male and female adults exhibit a greater BP response to large and smaller muscle-mass dynamic exercise than their younger counterparts (Julius et al., 1967; Martin et al., 1991; Ogawa et al., 1992; Stratton et al., 1994; Daida et al., 1996; Fisher et al., 2007; Sabbahi et al., 2018; Trinity et al., 2018). Although increases in resting BP with aging may influence these responses (Bassett et al., 1998), they are also observed in healthy, normotensive, and highly fit older males and females (Ogawa et al., 1992). For example, an early study, including 10,269 healthy participants with no history of hypertension (18–79 years, 7863 males and 2406 females), showed that peak changes in systolic and diastolic BP during maximal treadmill exercise testing were significantly higher in older males and females compared to their young counterparts (Daida et al., 1996). Males generally had higher BP responses across all decades, but the age effect appeared to accelerate in females after midlife (Daida et al., 1996). Similar findings were observed in a more recent study, including 2,736 individuals (20–79 years, 1525 males, and 1211 females) free of cardiovascular risk factors (e.g., hypertension and diabetes) (Sabbahi et al., 2018). Furthermore, compared with exercise-trained young male and female adults, exercise-trained older males and females also exhibit an elevated BP response to dynamic maximal exercise, with a more dramatic difference observed in the females (Ogawa et al., 1992). Indeed, females appear to experience a greater magnitude of change in resting and exercise BP with age (Martin et al., 1991; Ogawa et al., 1992; Narkiewicz et al., 2005). These studies strongly suggest that aging enhances the BP response to acute exercise, with the trajectory of these changes influenced by sex.

## Effects of aging on the exercise pressor reflex

The following section reviews the current evidence to decipher the contradictory results regarding the role of the exercise pressor reflex in mediating an exaggerated BP response to exercise with advancing age. Due to the limited space and studies available, we primarily focus on human studies attempting to isolate the reflex component of the exercise pressor response. We have organized the discussion based on the direction of the BP response (primary outcome) in older compared to younger adults and include supporting material on muscle sympathetic nerve (MSNA, secondary outcome) when available. See Table 1 for additional details on studies. A caveat with the studies presented below is that they mainly utilize low to moderate-intensity exercise modalities involving unilateral movement of smaller muscle mass (i.e., handgrip). Thus, although they are standard and well-controlled models providing critical insight, consideration must be taken that the mechanical and metabolic exercise stimulus is likely lower than that achieved during bilateral whole-body submaximal and maximal exercise. Furthermore, the majority of work has focused on the effect of aging on the metaboreflex and not the mechanoreflex component of the exercise pressor reflex. Thus, this is an important consideration when interpreting the studies and an avenue for future research.

**TABLE 1:** Overview of primary studies discussed.

1st author (year)	Methodology	Participants/animals	Key findings
Markel et al. (2003)	RHG (30% MVC) with incremental ischemia starting at min 2 of exercise and lasting for 5 min (+10 mmHg/min)	Healthy older [*n* = 7 (3 females), 65 years] vs. young [*n* = 6 (2 females), 24 years) adults	Blunted MAP, MSNA, and mean blood velocity in the exercising arm responses to RHG in the older group. Lower venous lactate and H+ in the exercising arm in the older group. Effects were mainly significant at higher levels of ischemia
Caron et al. (2018)	Electrically induced fatigue (EIF) muscle contraction of the hindlimb and chemical activation of metabosensitive afferents with KCI and LA	48 male rats evenly distributed in 4 age groups: 3, 6, 12, and 20 months. 21 exercise-trained male rats aged 12 and 20 months	Blunted metabosensitive afferent activity (recorded from the tibialis anterior and soleus muscles) in response to high but not low-level chemical activation in rats aged 12 and 20 months and to EIF in rats aged 20 months. No effect of exercise training
Momen et al. (2004)	Protocol 1: IHG (40% MVC) to fatigue + PEI. Protocol 2: Intermittent IHG (15 sec) at graded intensity (10%–70% MVC)	Healthy older [*n* = 7 (3 females), 65 years) vs. young [*n* = 9 (4 females), 25 years) adults	Similar MAP and HR during fatiguing and intermittent IHG and PEI between groups. Higher renal vascular resistance index during IHG in the older group
Ng et al. (1994)	IHG (40% MVC) to fatigue + PEI	Healthy older [*n* = 15 (7 females), 60–74 years) vs. young [*n* = 15 (7 females), 19–30 years) adults	Similar MAP response to fatiguing IHG and PEI between groups. Smaller increase in HR and relative MSNA in the older group
Houssiere et al. (2006)	IHG (30% MVC) + PEI performed with and without isocapnia hypoxia in random order	Sedentary but otherwise healthy older (OA, *n* = 12 (3 females), 55 years] vs. physically active healthy young [YA, *n* = 12 (2 females), 22 years] adults	Similar SBP and DBP but smaller HR and MSNA responses during IHG in the older group. Lower MSNA but similar SBP, DBP, and HR responses to PEI in the older group
Greaney et al. (2013)	IHG (30% MVC) + PEI	Healthy older (*n* = 10, 59 years) vs. young (*n* = 10, 24 years) male adults	Similar MAP and MSNA responses to IHG and PEI between groups. In a subset (*n* = 5 per group), lactate, potassium, and H+ increases during IHG were not different between groups
Sidhu et al. (2015)	Rhythmic single-leg knee extensor exercise (15 W, 30 W, and 80% of W max) under control conditions and with lumbar intrathecal fentanyl	Healthy older (*n* = 9, 68 years) vs. physically active young (*n* = 9, 24 years) male adults	Similar reduction In MAP, smaller reductions in CO (−17% vs. −5%), but a greater increase in leg vascular conductance (+11 vs. −8%) with fentanyl blockade in the older compared to the young group
D'Souza et al. (2023)	Incremental IHG to fatigue (+10% MVC/min) + PEI	Healthy older male (*n* = 11, 71 years) and female (*n* = 12, 70 years) adults vs. healthy young male (*n* = 12, 26 years) and female (*n* = 13, 25 years) adults	Similar MAP but blunted MSNA response to PEI in older adults. Greater MAP and lower neural recruitment responses to IHG in older males vs. other groups. Blunted MSNA response to IHG in the older group
Milia et al. (2015)	RHG (30% MVC) + PEI	Healthy but physically inactive older [*n* = 22 (12 females), 69 years, VO2peak = 24 ml/min/kg] vs. young physically active [*n* = 20 (10 females) 27 years, VO2peak = 45 ml/min/kg] adults	Exaggerated MAP response to PEI. Blunted and exaggerated CO and SVR response during PEI, respectively, in the older vs. younger group. RHG data not reported
Schneider et al. (2018)	IHG (30% MVC) + PEI. Before and after beetroot juice intervention	Healthy older [OA, *n* = 13 (6F), 67 ± 1 years] vs. young [YA, *n* = 10 (5F), 25 ± 1 years) adults. No regular physical activity in either group	Exaggereated SBP but not DBP response to PEI in older adults, similar trend seen during IHG, but not significant. Increasing plasma nitrate following 1 month of beetroot juice intervention attenuated SBP, DBP and MAP responses to PEI but not IHG vs. placebo trial
Hasegawa et al. (2021)	Primary protocol: RHG performed with and without muscle ischemia	Healthy older [OA, *n* = 23 (14 females), 69 years] vs. middle-aged [MA, *n* = 23 (12 females), 47 years] vs. young [YA, *n* = 26 (12 females), 22 years] adults. All participants reported being physically active	Exaggerated SBP response to RHG in older vs. young and middle-aged groups. Exaggerated SBP and DBP response to RHG + ischemia in older vs. young group. When pooling the data, age remained a significant predictor of BP response to RHG and RHG + ischemia
Wenner et al. (2022)	IHG (30% MVC) + PEI. Before and after 1 month estradiol intervention	Healthy older post-menopausal females (*n* = 13, 58 years, 9 years since menopause) vs. young pre-menopausal (*n* = 17, 22 years) females. No history of hormonal replacement therapy	Exaggerated MAP and MSNA response to IHG and PEI in post-menopausal vs. pre-menopausal females. Greater increase in TPR but similar CO during IHG in post-menopausal females. Higher and lower increases in TPR and CO, respectively, in post-menopausal females during PEI. 1 month of estradiol therapy decreased MAP response to IHG and PEI but affected MSNA only during IHG, not PEI
Park and Kim (2011)	Passive ankle dorsiflexion	Healthy post-menopausal (*n* = 10, 52 years) vs. pre-menopausal (*n* = 10, 45 years) females	Exaggerated MAP, blunted CO, and TVC during dorsal flexion in the post-menopausal vs. premenopausal females
Choi et al. (2012)	IHG (40% MVC) + PEI	Healthy post-menopausal (*n* = 15, 56 years) vs. pre-menopausal (*n* = 15, 37 years) females	In post-menopausal vs. pre-menopausal females exaggerated MAP response to IHG and PEI. Greater increase in SBP and DBP during PEI, but only SBP was different during IHG. Blunted increase in CO and reduction in TVC in post-menopausal vs. pre-menopausal females

### Blunted pressor reflex

The initial studies investigating the effect of age on the exercise pressor reflex suggested healthy older adults have a blunted reflex-evoked BP and MSNA response compared to young adults. For example, Markel et al. (2003) compared BP and MSNA responses to rhythmic handgrip exercise (30% maximal voluntary contraction; MVC) with incremental blood flow occlusion to enhance the metaboreflex in normotensive older adults with that in young adults (Markel et al., 2003). They found that the older group had an attenuated metaboreflex-evoked BP and MSNA, which was evident mainly when the highest level of blood flow occlusion was achieved. In line with these findings, a recent animal study showed that the responsiveness of metabosensitive thin afferents to chemical activation (potassium chloride and lactic acid) and electrically induced fatiguing rhythmic muscle contractions is lower in older compared to young male rats (Caron et al., 2018). However, this study only measured afferent responsiveness; thus, the BP effects are unknown. Together, these studies suggest that the metabosensitive component of the exercise pressor reflex is attenuated with aging.

### Preserved pressor reflex

Several studies also suggest that aging does not alter the exercise pressor reflex. Two early studies reported similar metaboreflex-evoked BP and MSNA responses following fatiguing isometric handgrip exercise (40% MVC) in healthy older and young adults (Ng et al., 1994; Momen et al., 2004), with no observed sex differences (Ng et al., 1994). Consistent with these findings, Houssiere et al. (2006) also found a preserved metaboreflex evoked BP response following isometric handgrip exercise (30% MVC) in healthy middle-aged compared to young adults (Houssiere et al., 2006). However, in line with Markel et al. (2003) mentioned above, this study found a blunted metaboreflex evoked MSNA response in the older group. This effect remained significant after normalizing for baseline MSNA burst frequency, which was nearly double in the older group. Greaney et al. (2013) also provided evidence of a preserved reflex function in middle-aged adult males. This study found similar BP and MSNA responses to isometric handgrip exercise (30% MVC) and isolated metaboreflex activation (PEI) in middle-aged compared to young adult males. More recently, D'Souza et al. (2023) evaluated potential age and sex interactions on the exercise pressor response and found that despite an elevated BP response to incremental (+10% MVC/min) isometric handgrip exercise in older males compared to young males and young and older females, the metaboreflex (PEI) evoked BP response was similar across groups. Conversely, in line with Markel et al. (2003) and Houssiere et al. (2006), both exercise and metaboreflex-evoked MSNA responses (both burst frequency and burst incidence) were significantly blunted in older compared to young adults. Finally, Sidhu et al. (2015) provided compelling and the only evidence of a preserved reflex during large muscle mass dynamic exercise using lumbar intrathecal fentanyl (Sidhu et al., 2015). Specifically, they found that fentanyl blockade similarly reduced BP during graded intensities (15 W, 30 W, and 80% of watt max) rhythmic single-leg knee extensor exercise in young and older males. Collectively, these studies provide compelling evidence of a preserved reflex evoked BP response to exercise with advancing age. These studies also highlight a potential divergence between the metaboreflex-evoked BP and MSNA response in older adults.

### Exaggerated pressor reflex

Studies have also provided convincing evidence of an exaggerated exercise pressor reflex in older adults compared to their young counterparts (Choi et al., 2012; Milia et al., 2015; Schneider et al., 2018; Hasegawa et al., 2021; Wenner et al., 2022). For example, studies have shown that the metaboreflex-evoked BP response following rhythmic (30% MVC) and isometric (30% MVC) handgrip exercise was significantly greater in older than in younger adults (Choi et al., 2012; Milia et al., 2015; Schneider et al., 2018). Interestingly, 4 weeks of nitrate supplementation (beetroot) was found to attenuate this BP response, indicating a role played by nitric oxide bioavailability (Schneider et al., 2018). Two of these studies compared sedentary older adults with young individuals reported to be quite physically active, which could have influenced the results (Milia et al., 2015; Schneider et al., 2018). However, an exaggerated reflex has also been demonstrated in physically active older adults. Specifically, one study showed that physically active older adults exhibited a greater BP response during ischemic low-intensity rhythmic handgrip exercise than physically active young and middle-aged adults (Hasegawa et al., 2021). Notably, age remained a significant predictor of the BP response when pooling the data. This study also compared BP responses to passive hand movement to evaluate the effect of age on the mechanoreflex and found no significant differences between age groups (Hasegawa et al., 2021). Although these findings suggest preserved mechanoreflex sensitivity, it is still plausible that the mechanoreflex is augmented during exercise as an ischemic milieu can sensitize mechanosensitive afferent activity (Rotto and Kaufman, 1988; Adreani and Kaufman, 1998; Cui et al., 2008).

## Consideration and future direction

When comparing study outcomes, it is essential to consider the differences in methods, protocols, and participant characteristics. Interestingly, sex was not equally represented in the studies mentioned, possibly contributing to variable findings from study to study (Table 1). Other plausible explanations for variability between study outcomes include the age of participants (e.g., middle *versus* older adult group), their physical activity or fitness levels, mode of exercise or limb involved (e.g., rhythmic *versus* isometric and leg *versus* arm exercise), or intensity at which the exercise was being performed. It is also worth considering that the effect size of aging on the exercise pressor reflex may be on the smaller side, requiring larger sample sizes and/or a greater exercise stimulus (e.g., intensity, muscle mass engaged, or modality).

### Potential role of skeletal muscle alterations

One possible explanation for study divergence warranting discussion is the magnitude of age-related alterations in skeletal muscle characteristics. Significant structural and functional skeletal muscle changes occur with aging [e.g., metabolism, fiber type, mass, and quality (i.e., force generating capacity per unit cross-sectional area)], contributing to decreased physical strength and metabolic alterations (e.g., insulin resistance) (Clement, 1974; Proctor et al., 1995; Kent-Braun et al., 2002; Purves-Smith et al., 2014; Liu and Zhu, 2023). The magnitude of both components of the exercise pressor reflex (i.e., metaboreflex and mechanoreflex) is heavily influenced by the amount of muscle mass engaged or tension produced, the composition of muscle fibers, and the resultant metabolic milieu during muscle contraction (Lind and McNicol, 1967; McCloskey and Streatfeild, 1975; Seals and Victor, 1991; Wilson and Mitchell, 1995; Estrada et al., 2020). Thus, it is possible that differences in structural and functional muscle characteristics with aging and between sexes could modulate reflex function. For example, in the study by D’Souza et al. (2023) a positive correlation (R = 0.204) was found between the absolute MVC (kg) attained and the peak exercise BP across groups. While absolute MVC was similar between young and older males, it was significantly lower in the older compared to young females. The authors noted this could have masked an effect of age in the females. Indeed, when comparing young and older females with similar MVC, they found a tendency toward a higher BP response to fatiguing handgrip exercise in older females. However, these analyses were underpowered. A recent study highlights the importance of considering absolute muscle strength when comparing the exercise pressor response between groups (Notay et al., 2018). Thus, future studies should consider this in the study design, analyses, and interpretation.

### Potential role of reflex arc alterations

It is important to consider that aging may affect the exercise pressor reflex arc at multiple levels, including the concentration of stimulants present around the afferent endings (e.g., metabolites and inflammatory products), afferent sensitivity (e.g., receptor or channel expression or co-expression), central processing in the brainstem, as well as the efferent activity. Although one study suggested a lower metabolite production (e.g., lactic acid and H+) during muscle contraction could contribute to the blunted metaboreflex observed in older compared to younger adults (Markel et al., 2003), other studies suggest metabolite production (e.g., lactic acid, pH, potassium, ADP, inorganic phosphate) is not affected by aging (Greaney et al., 2013; Trinity et al., 2018). Moreover, Trinity et al. (2018) observed no sex difference in metabolite production in older adults, despite a greater BP response in older females compared to older males and young adults. However, it is essential to note that in these studies, only a few out of the many stimulants thought to contribute to evoking the exercise pressor reflex have been considered (Tallarida et al., 1979; Rybicki et al., 1985; Stebbins et al., 1986; Rotto and Kaufman, 1988; Rotto et al., 1989; Rotto et al., 1990; Pan et al., 1993; Fadel et al., 2003; Cui et al., 2007; Ducrocq and Kaufman, 2020). Interestingly, data from animal studies suggest aging directly alters the muscle afferent activity response to mechanical and metabolic stimuli (Taguchi and Mizumura, 2011; Caron et al., 2018). However, this may depend on the type of stimuli applied. For example, one study showed a blunted response of metabosensitive group III and IV afferents to potassium chloride, lactic acid, and electrically induced fatiguing rhythmic muscle contractions in older compared to young male rats (Caron et al., 2018). Conversely, another study showed a similar group IV afferent response to pH 5.5, ATP, and bradykinin but a greater response to mechanical stimuli in older compared to younger male rats (Taguchi and Mizumura, 2011). Other studies indicate that afferent and efferent C-fibers undergo morphological and structural changes with advancing age, contributing to abnormal peripheral nerve discharge characteristics (Bergman and Ulfhake, 1998; Ceballos et al., 1999; Namer et al., 2009; Namer, 2010). These findings highlight several potential functional consequences of age-related skeletal muscle and reflex arc alterations. However, additional studies are needed.

### Potential role of insulin resistance

Another notable change with aging is increased insulin resistance, which appears to influence the exercise pressor reflex (Brett et al., 2000; Holwerda et al., 2016; Hotta et al., 2020). For example, a recent study showed that insulin-resistant older adults have a greater metaboreflex-evoked BP response than insulin-sensitive older adults (Hotta et al., 2020). Moreover, other studies have found a positive correlation between BP and MSNA responses to exercise and markers of insulin resistance (Brett et al., 2000; Holwerda et al., 2016). The mechanism(s) mediating the insulin resistance effect on the exercise pressor reflex remains to be determined; however, emerging animal data suggest insulin directly enhances the responsiveness of peripheral thin fiber afferents to mechanical and chemical stimuli (Hotta et al., 2019; Hori et al., 2022). Moreover, insulin has strong central autonomic effects that may also be involved (Morgan et al., 1993; Muntzel et al., 1994; Estrada et al., 2023). Conversely, glucose may be less important (Huo et al., 2020). These findings highlight the importance of considering insulin resistance status and circulating insulin levels when studying the exercise pressor reflex, especially in insulin-resistant prone older adults.

## Perspectives

**FIGURE 1:**
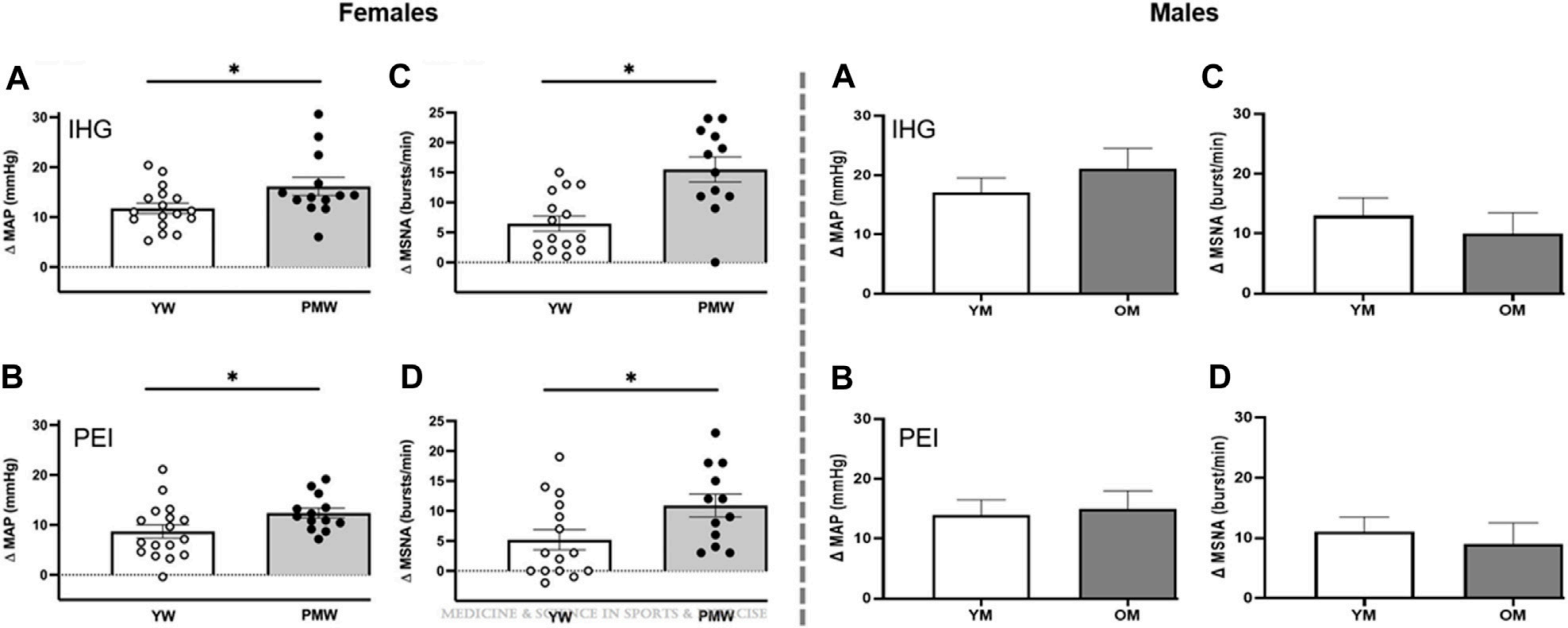
Mean arterial pressure (MAP, **(A,B)** and muscle sympathetic nerve activity (MSNA, **(C, D)** responses to isometric handgrip exercise (IHG, top) performed at 30% maximal voluntary contraction and post-exercise ischemia (PEI; bottom) in older post-menopausal women (PMW) compared to younger pre-menopausal women (YW, left panels) and in older men (OM) compared to young men (YM, right panels). Modified figures from Wenner et al. (2022) and Greaney et al. (2013), with permission.

Appropriate autonomic adjustments are critical for initiating and sustaining exercise by increasing BP and facilitating the redistribution of blood flow and oxygen delivery to meet the metabolic demands of the active skeletal muscle. Aging may negatively affect this ability, as highlighted by an exaggerated BP response and attenuated maximal oxygen uptake (Ogawa et al., 1992; Fitzgerald et al., 1997; Seals et al., 2001; Currie et al., 2017). Given the importance of autonomic adjustments and the significant contribution of the exercise pressor reflex in these adjustments, it is difficult to reconcile the conflicting findings related to the effect of aging on the reflex-mediated BP response during exercise. However, the reviewed literature hints at a possibly greater impact of aging on the exercise pressor reflex in females than males. Specifically, the majority of studies indicating an exaggerated reflex response include exclusively or a greater representation of females. Conversely, most studies showing a blunted or preserved reflex have exclusively included males or a greater representation of males. Nonetheless, the protocol used requires careful consideration. Indeed, when comparing BP and MSNA responses to PEI following isometric handgrip, an exaggerated response is only observed in post-menopausal females, not in males (Figure 1) (Greaney et al., 2013; Wenner et al., 2022). Conversely, when comparing BP and MSNA responses to PEI following fatiguing rhythmic or isometric handgrip, there are no differences across age or sex (Ng et al., 1994; D'Souza et al., 2023). Therefore, considering and comparing reflex responses to different exercise stimuli will be necessary for future studies to better understand the significance of these findings.

### Altered reflex hemodynamic responses

Furthermore, evaluating the impact of reflex activation on the components determining BP during exercise may provide additional insight. As defined by Ohm’s law, the BP response to exercise is the product of cardiac output and total peripheral resistance (TPR). Aging results in a dramatic shift in the contribution of these two components to BP in both males and females, which is exacerbated during exercise. Specifically, cardiac output contributes less to BP with increasing age due mainly to decreased stroke volume and heart rate (Ogawa et al., 1992; Stratton et al., 1994; Seals et al., 2001; Tanaka and Seals, 2003; Seffrin et al., 2023). Conversely, TPR contributes more to BP with increasing age due to greater vasoconstrictor tone (i.e., increased sympathetically mediated vasoconstrictor activity, decreased arterial compliance, and vasodilator capacity) (Gerhard et al., 1996; Dinenno et al., 2001; Seals et al., 2001; Singh et al., 2002; Koch et al., 2003; Trinity et al., 2018; Hearon et al., 2020). In line with this, the reflex-evoked BP response is primarily driven by increased peripheral vasoconstrictor tone in both male and female older adults (Stratton et al., 1994; Sidhu et al., 2015; Wenner et al., 2022; D'Souza et al., 2023). Interestingly, inhibiting muscle afferent feedback from the contracting muscles during submaximal single-leg kicking reduces BP similarly in young and older adult males but dramatically improves systemic and leg vascular conductance in older but not younger males (Sidhu et al., 2015). Although only shown in males, these findings may implicate a significant role played by group III and IV muscle afferents in limiting skeletal muscle blood flow during exercise in older adults. Theoretically, this would augment reflex activation and reduce oxygen delivery to the exercising muscles, especially during activities that dramatically challenge cardiovascular regulation (e.g., large muscle mass/whole body and higher intensity).

### Sex-specific impacts

The literature reviewed highlights a complex relationship between age and sex regarding the reflex control of cardiovascular responses to exercise. Sex is a well-known, crucial biological determinant of neurovascular control (Ettinger et al., 1996; Joyner et al., 2015; Klassen et al., 2021). Indeed, while young females exhibit lower resting and exercising BP and MSNA than males, females exhibit a greater magnitude increase in these outcomes with age compared to males (Martin et al., 1991; Ogawa et al., 1992; Ettinger et al., 1996; Narkiewicz et al., 2005; Jarvis et al., 2011; Ji et al., 2020; Keir et al., 2020). Current evidence supports that the exaggerated exercise BP in females is strongly related to menopausal changes and its effect on the exercise pressor reflex. For example, the BP response to passive ankle dorsiflexion, a technique used to evoke the mechanoreflex in humans, was significantly greater in middle-aged post-menopausal females compared to middle-aged pre-menopausal females (Park and Kim, 2011). These findings were later extended to include the metaboreflex with two separate investigations showing exaggerated BP and MSNA response to isometric handgrip exercise and PEI in middle-aged post-menopausal females compared to younger pre-menopausal females (Choi et al., 2012; Wenner et al., 2022). These findings are consistent with other studies showing sex-specific changes in exercise BP (Trinity et al., 2018) and collectively highlight that mechano- and metaboreflex activity is enhanced after menopause. See recent review for additional details (Smith et al., 2019).

### Role of menopause

Although speculative, the augmented exercise pressor reflex observed in post-menopausal females may impair contracting muscle blood flow, which could further amplify reflex activation, ultimately resulting in a positive feedback loop. This hypothesis is supported by studies showing that post-menopausal females exhibit a greater decrease in peripheral vascular conductance during mechano- and metaboreflex activation and an excessive increase in sympathetically mediated vasoconstriction in the exercising muscle (Fadel et al., 2004; Park and Kim, 2011; Choi et al., 2012). Interestingly, estrogen replacement therapy is effective in attenuating the augmented metaboreflex and sympathetically mediated vasoconstriction in post-menopausal females, suggesting that these age-related changes are modifiable and due to the estrogen loss with menopause (Fadel et al., 2004; Wenner et al., 2022). These finding may not be surprising when considering that estrogen directly affects the neural pathways responsible for evoking the exercise pressor reflex and have strong vasodilatory effects that indirectly can modulate the reflex by affecting the metabolic milieu in the active skeletal muscle (Gilligan et al., 1994; Schmitt et al., 2006; Koba et al., 2012; Samora et al., 2019).

### Role of sex hormones

The interaction between age and sex is increasingly recognized under resting conditions. However, the translation of this to exercise remains relatively understudied. Notably, we know much more about female sex hormones and their impact on the exercise pressor reflex than we do about male sex hormones (i.e., testosterone). Indeed, although studies have demonstrated a significant relationship between testosterone levels and resting BP and MSNA (Khaw and Barrett-Connor, 1988; Fogari et al., 2005; Carter et al., 2012), surprisingly little is known regarding its impact on neural cardiovascular control during exercise. Interestingly, a recent study implicates testosterone levels as a significant predictor of the exercise pressor response in males (D'Souza et al., 2023). Thus, determining whether age-related declines in testosterone and testosterone treatment similarly affect the exercise pressor reflex as observed with estrogen in females will provide critical insight. Indeed, we hope future studies continue to uncouple the effect of aging and its complex interaction with sex and associated hormones on the exercise pressor reflex.
